# ENTPD8 overexpression enhances anti-PD-L1 therapy in hepatocellular carcinoma via miR-214-5p inhibition

**DOI:** 10.1016/j.isci.2025.111819

**Published:** 2025-01-16

**Authors:** Si-qi Zhao, Min-jie Chen, Fei Chen, Zhao-feng Gao, Xiao-ping Li, Ling-yu Hu, Hai-ying Cheng, Jin-yan Xuan, Jian-guo Fei, Zheng-wei Song

**Affiliations:** 1 Department of Surgery, the Second Affiliated Hospital of Jiaxing University, Jiaxing, Zhejiang, China; 2 Department of General Practice, the Second Affiliated Hospital of Jiaxing University, Jiaxing, Zhejiang, China

**Keywords:** molecular biology, cell biology, cancer

## Abstract

Hepatocellular carcinoma (HCC) is a leading cause of cancer-related deaths globally, with poor prognosis due to late diagnosis and limited treatment options. In this study, we evaluated the expression of ectonucleoside triphosphate diphosphohydrolase 8 (ENTPD8) in HCC tissues and its clinical significance. Immunohistochemistry, The Cancer Genome Atlas (TCGA) data, and single-cell expression analysis revealed reduced ENTPD8 levels in liver cancer compared to adjacent tissues, with ENTPD8 primarily expressed in tumor cells within the tumor tissue. *In vitro* assays demonstrated that ENTPD8 inhibits HCC cell proliferation, invasion, and migration. Mechanistically, ENTPD8 regulates programmed death-ligand 1 (PD-L1) expression through miR-214-5p modulation. *In vivo*, ENTPD8 overexpression combined with anti-PD-L1 treatment enhanced therapeutic efficacy in HCC mouse models. These findings suggest that ENTPD8 may serve as a prognostic marker and therapeutic target for HCC, offering potential strategies for improving treatment outcomes.

## Highlights

• ENTPD8 expression is reduced in HCC tissues and correlates with prognosis
• ENTPD8 inhibits HCC cell proliferation, invasion, and migration *in vitro*
• ENTPD8 downregulates miR-214-5p to modulate PD-L1 expression in HCC
• ENTPD8 overexpression with anti-PD-L1 improves therapeutic efficacy in HCC

## Graphical abstract

**Figure:**
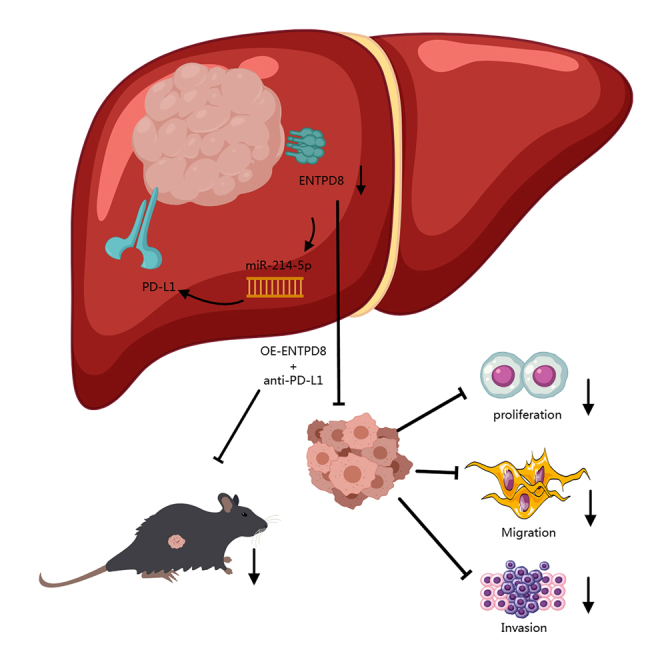

## Introduction

According to global cancer statistics, there were approximately 900,000 cases of hepatocellular carcinoma (HCC) in 2020, resulting in 830,000 deaths, making it the sixth most common cancer and the third leading cause of cancer-related deaths worldwide.^1^ HCC is closely associated with liver diseases such as cirrhosis, chronic hepatitis B, and precancerous lesions.^2^^,^^3^ The systemic treatment options for primary liver tumors have advanced significantly in recent years.^4^^,^^5^ Traditionally, liver cancer treatment relied on surgery, local ablation, and chemotherapy, though with limited efficacy. With deeper insights from molecular biology research, various targeted therapies, such as sorafenib and lenvatinib, have been developed, significantly improving survival rates for patients with advanced liver cancer. Additionally, immune checkpoint inhibitors (e.g., programmed cell death protein 1 [PD-1]/programmed death-ligand 1 [PD-L1] inhibitors) have become a focus, harnessing the patient’s immune system to fight the tumor. Combination therapy strategies are also emerging.^6^ with the combination of targeted drugs and immunotherapy showing synergistic effects that further extend patient survival. Nevertheless, due to HCC’s extremely aggressive behavior and resistance to conventional therapy, there is still an urgent need to optimize the diagnosis, treatment, and prognostic management of HCC.^7^

ENTPD8 (ectonucleoside triphosphate diphosphohydrolase 8) is a membrane protein belonging to the E-NTPDase family, possessing five transmembrane domains.^8^ ENTPD8 is primarily expressed in endothelial cells, hepatocytes, lymphocytes, and macrophages. Its active site is situated at the extracellular end of the protein, where it acts on extracellular nucleotides and nucleoside triphosphates. The protein features a long extracellular domain at the N terminus and a zinc-containing core domain at the C terminus.^8^^,^^9^^,^^10^ The primary physiological function of ENTPD8 is to hydrolyze extracellular ADP and ATP into AMP and phosphate, thereby regulating extracellular nucleotide levels. This enzyme activity is crucial for controlling extracellular ATP concentration, which in turn significantly influences extracellular ATP signaling.^8^^,^^9^^,^^11^ Moreover, ENTPD8 is involved in biological processes such as cell adhesion, apoptosis, immune responses, and cell migration.^9^^,^^12^^,^^13^ However, its exact role in human cancer, especially in HCC, remains incompletely understood.

Tumor immunotherapy, particularly therapies related to PD-1/PD-L1, is currently one of the most promising and widely researched areas in the field of cancer treatment.^14^^,^^15^^,^^16^ PD-L1, an immune checkpoint molecule, typically plays a vital regulatory role between immune cells (such as T cells) and tumor cells.^17^^,^^18^^,^^19^ When tumor cells express PD-L1, it can bind to PD-1 on the surface of T cells, thereby suppressing T cell immune activity and promoting tumor evasion and growth.^20^^,^^21^ PD-L1 monoclonal antibodies block the binding of PD-L1 to PD-1, restoring T cell activity and enhancing their recognition and cytotoxicity against tumor cells.^22^^,^^23^ Currently, PD-L1 monoclonal antibodies are widely used in the treatment of various malignancies, including HCC, melanoma, non-small cell lung cancer, breast cancer, and head and neck cancer.^22^^,^^24^^,^^25^ Clinical trials have demonstrated the great potential of PD-L1 in improving patient survival rates and prolonging progression-free survival. However, resistance to PD-L1 monoclonal antibodies remains a significant challenge.^26^^,^^27^ During tumor progression, cancer cells may evade the therapeutic effects of PD-L1 antibodies by regulating PD-L1 expression. Additionally, an increase in other immune inhibitory cells, such as regulatory T cells, within the tumor microenvironment may contribute to the failure of PD-L1 monoclonal antibodies. Furthermore, tumor cells may enhance their resistance by expressing other immune inhibitory factors or reducing antigen presentation.^28^^,^^29^^,^^30^ Combining PD-L1 antibodies with other immunotherapeutic agents, such as CTLA-4 inhibitors, as well as with chemotherapy or targeted therapies, is an effective strategy to prolong efficacy and slow the development of resistance.^18^^,^^31^ However, the application of immune checkpoint inhibitors also faces many challenges, including drug resistance and adverse reactions (such as tinnitus and neurotoxicity).^32^^,^^33^ Therefore, key open questions in HCC immunotherapy, including preclinical studies, effective immunotherapies and immune-based combination therapies, response biomarkers, experimental therapies, and practical application experience of immune checkpoint inhibitors, need to be further explored and studied.^34^

This study aims to explore the expression and function of ENTPD8 in HCC and its potential role in immunotherapy. Our research aims to provide a target and strategy for HCC treatment, promoting the application of immunotherapy in HCC.

## Results

### The expression of ENTPD8 was decreased in HCC

**Figure 1:**
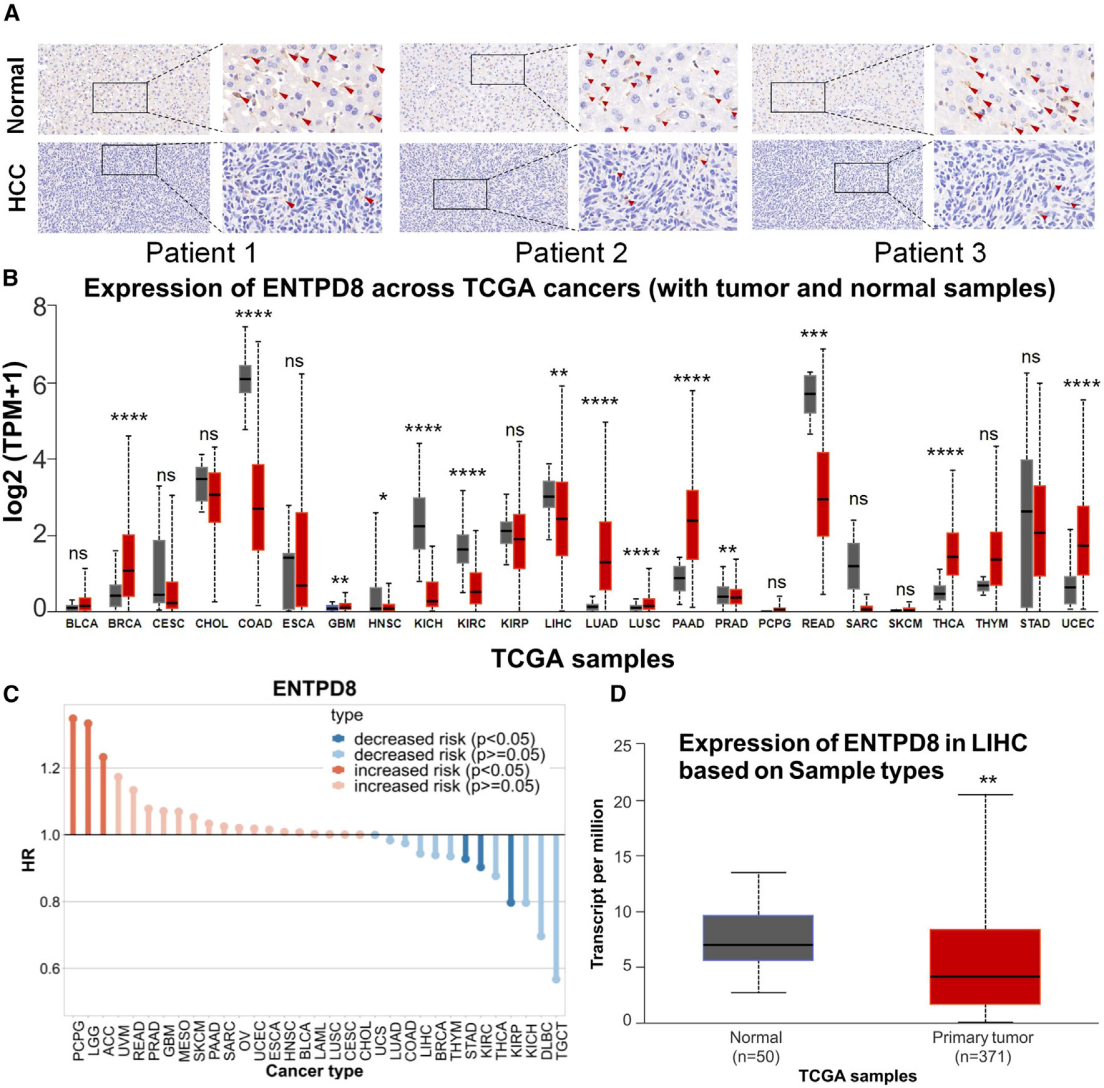
Expression profile of ENTPD8 in HCC (A) Immunohistochemistry results of ENTPD8 expression in the HCC and corresponding paracancer tissues. (B) Expression of ENTPD8 mRNA in different cancers and corresponding normal tissues. (C) Relationship between ENTPD8 expression and risk of different cancers. (D) The expression of ENTPD8 mRNA in HCC was significantly higher than that in normal liver tissue. Data are represented as mean ± SEM.

We performed immunohistochemistry on tumor tissues and corresponding adjacent tissues from three patients with HCC, showing a significant downregulation of ENTPD8 expression in HCC (Figure 1A). Subsequently, we used GTEx and UALCAN to analyze the expression of ENTPD8 in tumor tissues and normal tissues of different cancer types. We found significant differences in ENTPD8 gene expression among different cancers. ENTPD8 expression was lower in liver cancer, colorectal cancer, renal cancer, and gastric cancer compared to normal tissues, while it was higher in breast cancer, lung cancer, and pancreatic cancer, among others (Figure 1B). This suggests strong tumor specificity of ENTPD8 expression. Furthermore, we utilized the Tumor Immune Single-cell Hub 2 (TISCH) database to further analyze the relationship between ENTPD8 and the mortality risk of different cancer types, and the results indicate a trend of negative correlation between ENTPD8 expression and the mortality risk in patients with HCC, but it was worth noting that there was no statistical significance between the two (Figure 1C). The low expression of ENTPD8 in HCC tissues (Figure 1D) and its association with the risk of death in patients with HCC suggest that ENTPD8 may be a potential anti-cancer target in HCC. Therefore, we further analyzed the relationship between ENTPD8 expression and HCC using The Cancer Genome Atlas and UALCAN databases. The results showed that as the grade of liver cancer increased, the expression of ENTPD8 decreased (Figure S1A). Additionally, we demonstrated the relationship between ENTPD8 and different stages, races, genders, ages, and weights (Figures S1B–S1H).

### Single-cell level analysis and subcellular localization of ENTPD8

**Figure 2:**
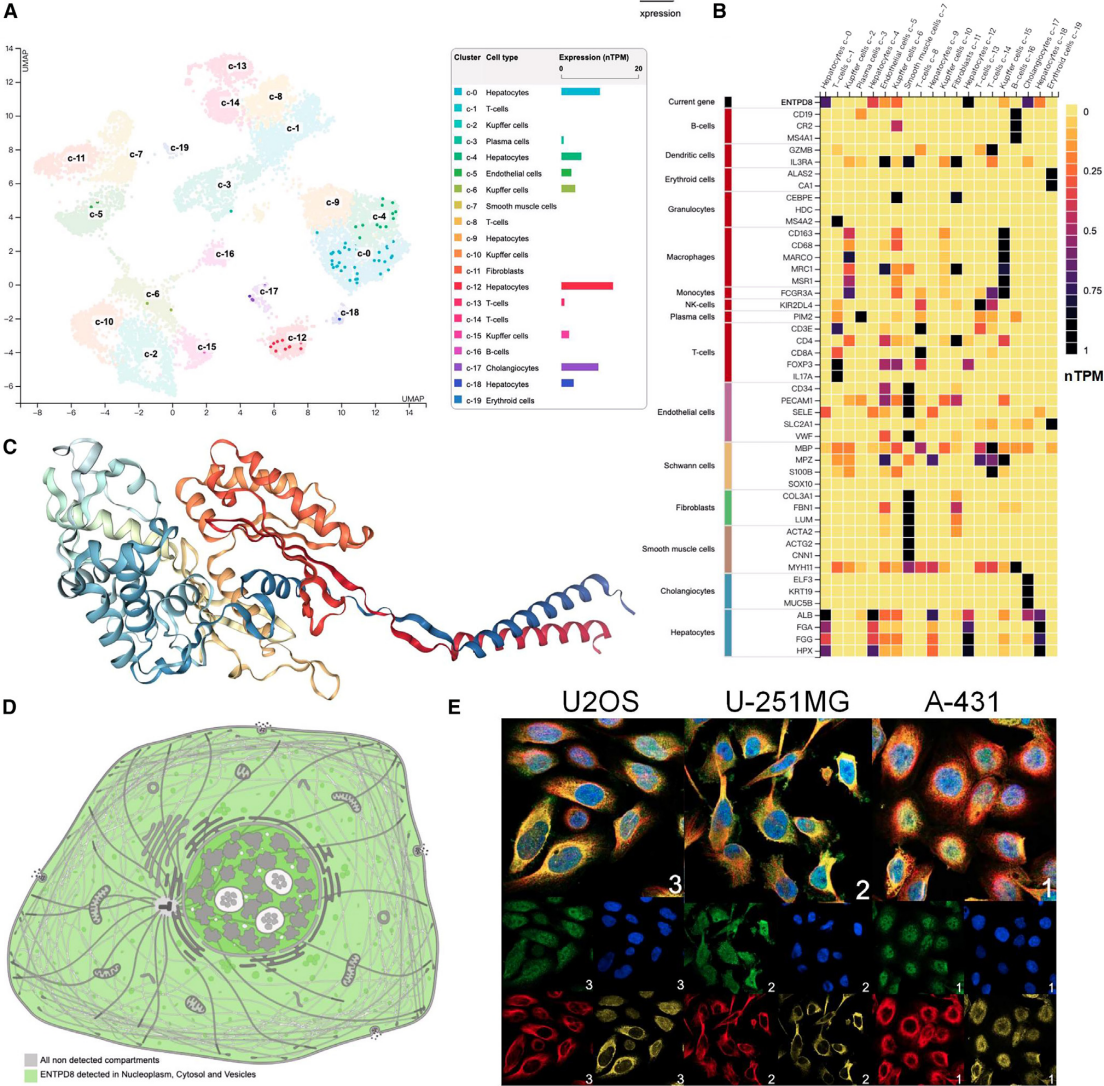
Single-cell level expression profile and subcellular localization of ENTPD8 (A) Uniform manifold approximation and projection (UMAP) map identifies 19 cell clusters in normal liver tissue. (B) Expression of ENTPD8 in different cells. (C) 3D structure diagram of ENTPD8. (D) Subcellular mapping of ENTPD8. (E) ENTPD8 staining in subcellular localization in U2OS, U-251MG, and A-431 cells.

**Figure 3:**
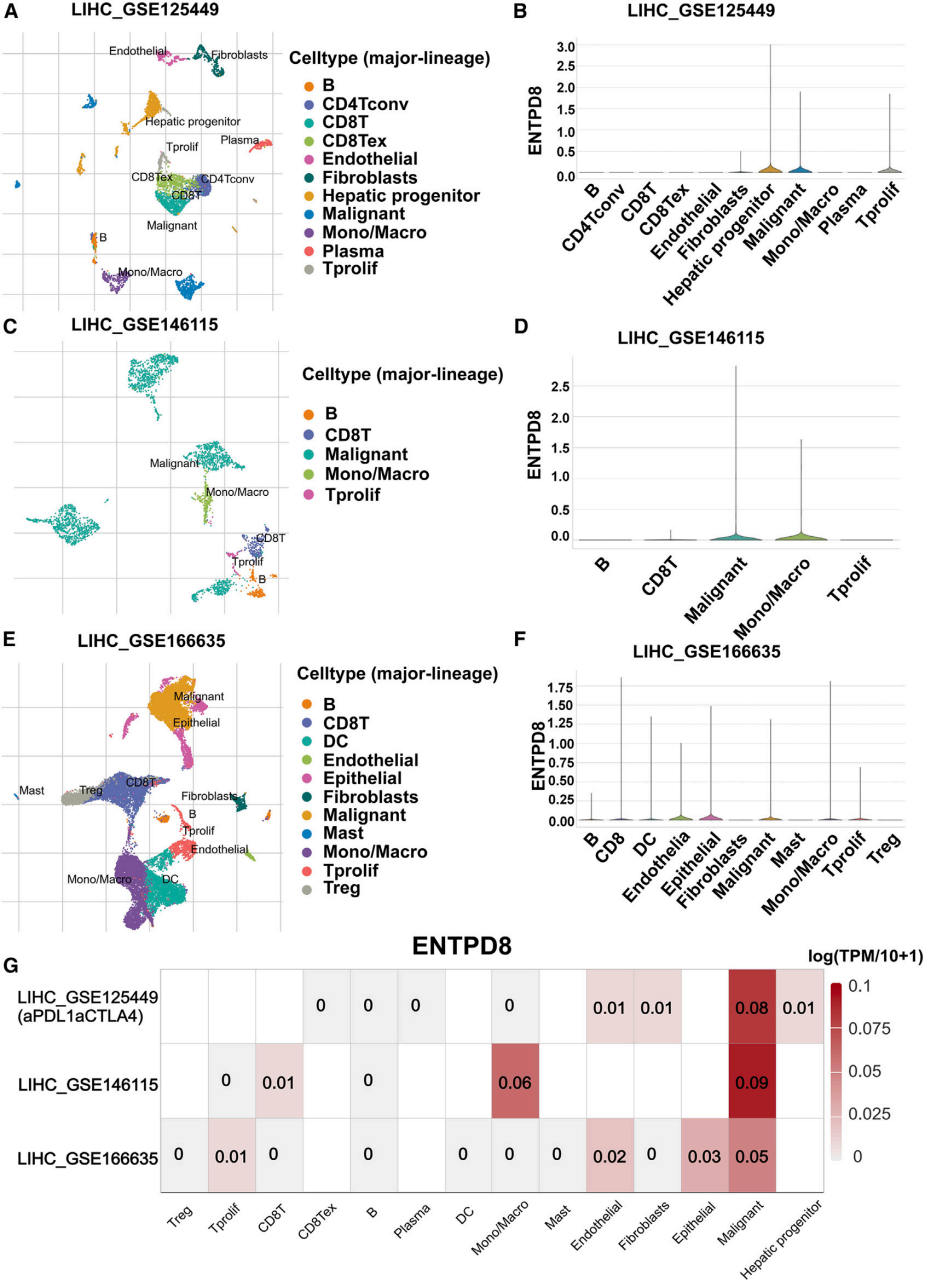
ENTPD8 was enriched in malignant cells of tumors based on scRNA-seq (A) UMAP map identifies 6 cell clusters that define the classification of specific gene signatures, including B cells, endothelial cells, fibroblasts, mono/macro cells, plasma cells, malignant cells, and T cells based on GSE125449. (B) The UMAP map and violin plot showing the expression of ENTPD8 in malignant cells based on GSE125449. (C) UMAP map identifies 6 cell clusters that define the classification of specific gene signatures, including B cells, mono/macro cells, malignant cells, and T cells based on GSE146115. (D) The UMAP map and violin plot showing the expression of ENTPD8 in malignant cells based on GSE146115. (E) UMAP map identifies 6 cell clusters that define the classification of specific gene signatures, including B cells, dendritic cells (DCs), endothelial cells, epithelial cells, fibroblasts cells, mast cells, mono/macro cells, malignant cells, T cells, and Treg based on GSE166635. (F) The UMAP map and violin plot showing expression of ENTPD8 in malignant cells based on GSE166635. (G) Single-cell expression of ENTPD8 in HCC.

To further explore the expression of ENTPD8, we examined its single-cell expression levels in normal liver using the Human Protein Atlas database. Single-cell sequencing results showed that hepatocytes in normal liver tissue were divided into five subgroups according to the expression levels of albumin, fibrinogen alpha, fibrinogen gamma, and hemopexin, and ENTPD8 was mainly expressed in these subgroups of hepatocytes, while it was less expressed in other cell types such as immune cells (Figures 2A and 2B). Additionally, we simulated the three-dimensional structure of ENTPD8 (Figure 2C). At the subcellular level, ENTPD8 was mainly detected in the nucleoplasm, cytosol, and vesicles (Figure 2D). Fluorescence staining of ENTPD8 was also observed in human epidermoid carcinoma cell line A-431, human glioblastoma cell line U-251MG, and human osteosarcoma cell line U-2 OS (Figure 2E). Furthermore, using the TISCH database, we analyzed the expression of ENTPD8 in single-cell data from liver cancer datasets GSE125449, GSE146115, and GSE166635, revealing that ENTPD8 is primarily expressed in HCC cancer cells (Figures 3A–3G).

### ENTPD8 knockdown promoted the proliferation, invasion, and migration ability of human HCC cell lines

**Figure 4:**
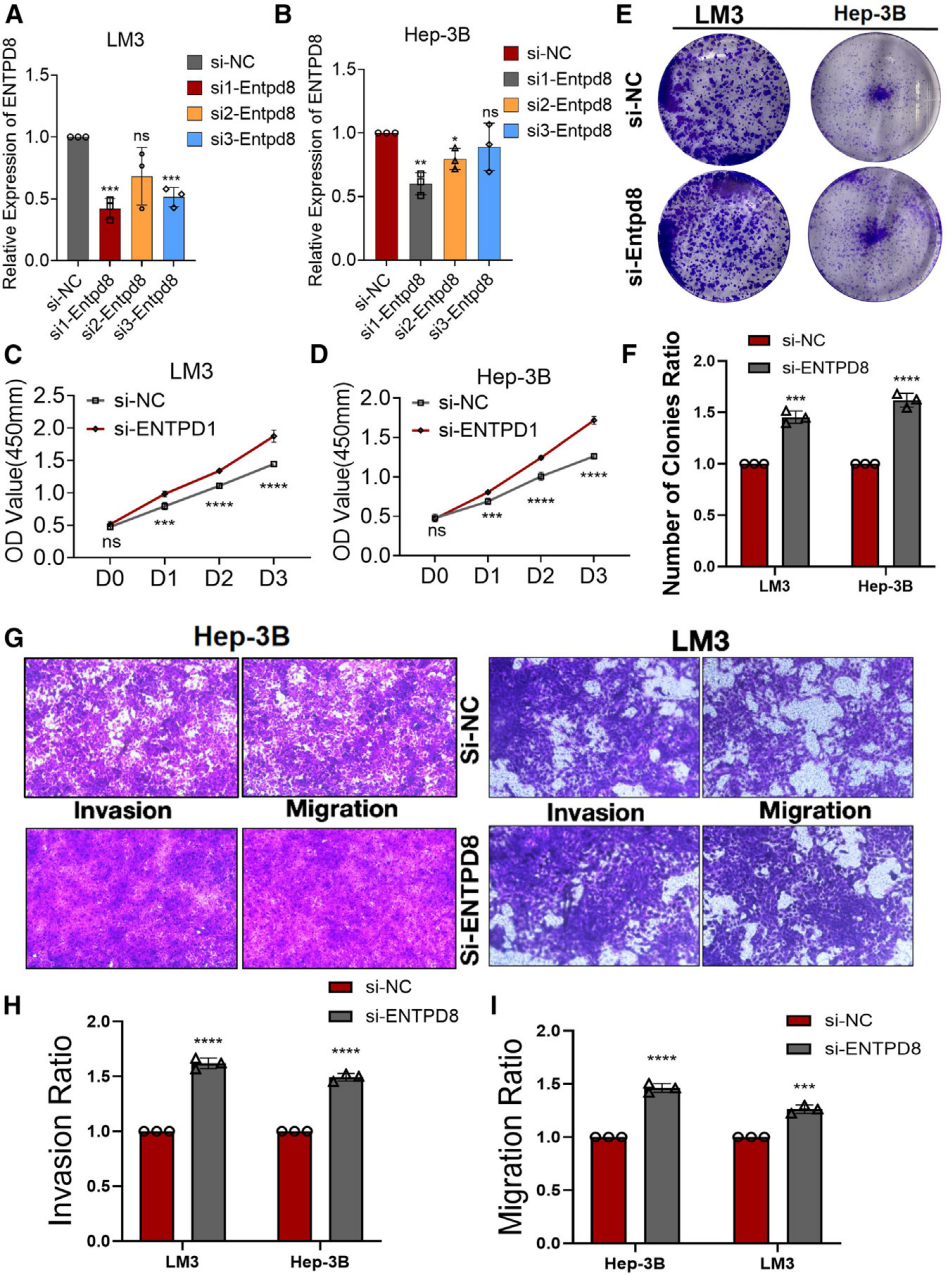
ENTPD8 knockdown promoted the proliferation, invasion, and migration ability of human HCC cell lines (A and B) Three siRNAs (si1, si2, and si3) were designed to silence ENTPD8 in HCC cells (Hep3B and LM3) and validated by qRT-PCR. (C and D) The growth curves of LM3 (C) and Hep3B (D) cells were plotted after transfection with si1-ENTPD8/si-NC based on CCK-8 assay. (E and F) Colony formation assays demonstrated that knockdown of ENTPD8 promoted the proliferation of LM and Hep3B cells. (G–I) Transwell experiment demonstrated that knockdown of ENTPD8 expression could effectively promote the migration and invasion ability of HCC cells. ∗, *p* < 0.05; ∗∗, *p* < 0.01; ∗∗∗, *p* < 0.001; ∗∗∗∗, *p* < 0.0001. Data are represented as mean ± SEM.

The aforementioned results indicated that ENTPD8 was mainly expressed in hepatoma cells, prompting us to further investigate the effects of ENTPD8 knockdown on the proliferation, invasion, and migration of HCC cells. We synthesized three small interfering (si)RNAs specifically targeting the ENTPD8 gene to modulate its expression within Hep3B and LM3 cell lines. Quantitative reverse-transcription PCR (qRT-PCR) analysis identified si1-ENTPD8 as the most effective in reducing ENTPD8 expression, which justified its selection for subsequent studies (Figures 4A and 4B). CCK8 assays showed a significant increase in cell proliferation in both Hep3B and LM3 cell lines following ENTPD8 knockdown (Figures 4C and 4D). Colony formation assays corroborated these findings, revealing similar increases in proliferation (Figures 4E and 4F). Additionally, transwell assays demonstrated that migration and invasion capabilities were markedly elevated after ENTPD8 reduction (Figures 4G–4I). These results support the hypothesis that ENTPD8 may possess potential anti-cancer properties in the context of HCC.

### Overexpression of ENTPD8 suppressed the proliferation, invasion, and migration ability of human HCC cell lines

**Figure 5:**
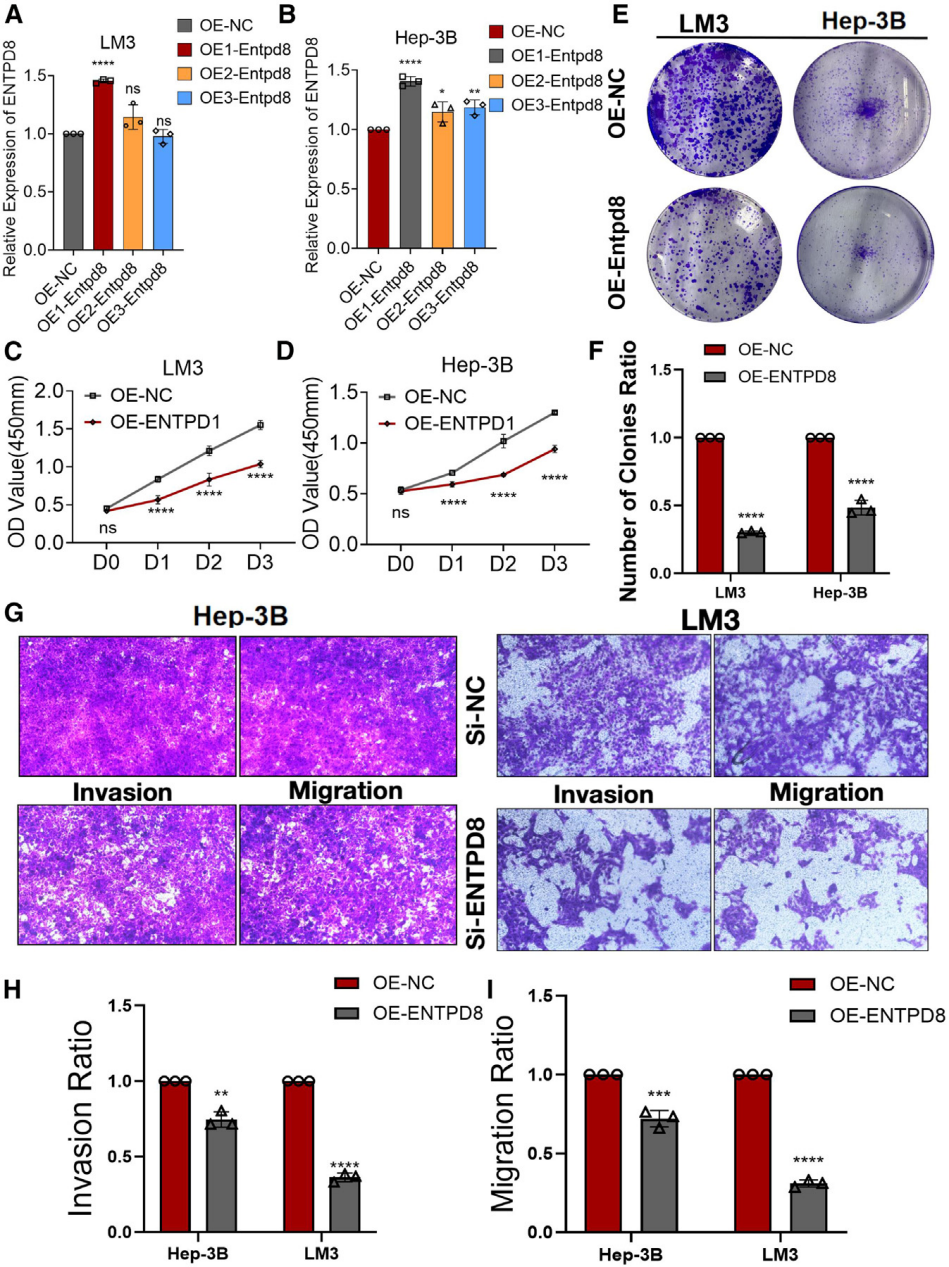
Overexpression of ENTPD8 inhibited proliferation, invasion, and migration of human HCC cell lines (A and B) qRT-PCR verified the efficiency of overexpression of ENTPD8 in LM3 and Hep3B cell lines. (C and D) The growth curves of LM3 (C) and Hep3B (D) cells were plotted after overexpression of ENTPD8 based on CCK-8 assay. (E and F) Colony formation assays demonstrated that overexpression of ENTPD8 inhibited the proliferation of LM3 and Hep3B cells. (G–I) Transwell experiment demonstrated that overexpression of ENTPD8 could effectively inhibit the migration and invasion ability of HCC cells. ∗, *p* < 0.05; ∗∗, *p* < 0.01; ∗∗∗, *p* < 0.001; ∗∗∗∗, *p* < 0.0001. Data are represented as mean ± SEM.

The results following ENTPD8 knockdown suggested that ENTPD8 inhibits HCC cell activity. To more precisely confirm its role, we upregulated ENTPD8 expression in HCC cells. We introduced three distinct ENTPD8-targeting lentiviruses into human HCC cell lines to elevate ENTPD8 expression. qRT-PCR results spotlighted the viral construct OE1-ENTPD8 as the most efficient at increasing ENTPD8 levels (Figures 5A and 5B). CCK8 proliferation assays revealed a significant reduction in HCC cell growth upon ENTPD8 overexpression (Figures 5C and 5D). This inhibitory effect on cell proliferation was further supported by colony formation assays, which suggested that ENTPD8 modulates cellular expansion (Figures 5E and 5F). Additionally, transwell assays showed a notable decrease in invasion and migration capabilities in Hep3B and LM3 cells with elevated ENTPD8 expression (Figures 5G and 5H). Together, these results highlight the potential of ENTPD8 expression as a factor in mitigating HCC progression.

### ENTPD8 can be used as a prognostic indicator in patients with HCC

The aforementioned results strongly indicate a potential inhibitory role of ENTPD8 in HCC. However, does the role of ENTPD8 in patients with HCC correspond to its cellular-level function? Therefore, we utilized the Kaplan-Meier plotter to investigate the association between ENTPD8 expression and patient prognosis. We obtained exciting analysis results. Patients with high ENTPD8 expression showed significantly better outcomes in overall survival (OS) (Figure S2A), recurrence-free survival (Figure S2B), progression-free survival (PFS) (Figure S2C), and disease-specific survival (Figure S2D) compared to patients with low ENTPD8 expression. Additionally, we preliminarily investigated the relationship between ENTPD8 expression and survival after immunotherapy in patients with pan-cancer (including bladder cancer, esophageal cancer, glioblastoma, hepatocellular carcinoma, head and neck squamous cell carcinoma, melanoma, non-small cell lung cancer, etc.). Surprisingly, the results indicate that patients with high expression of ENTPD8 have a better prognosis with immunotherapy. Although there was no statistically significant difference in OS between patients with high ENTPD8 expression and those with low expression after receiving anti-PD1 therapy (Figure S2E), the former showed significantly better PFS than the latter (Figure S2F). Importantly, patients with high ENTPD8 expression appear to show significantly better outcomes in both OS and PFS after anti-PD-L1 treatment compared to patients with low expression (Figures S2G and S2H).

Although the aforementioned results suggest that there may be a certain correlation between ENTPD8 expression and the efficacy of immunotherapy, it is noteworthy that these results cannot exclude the survival benefit brought by high ENTPD8 expression itself. Therefore, these results only indicate that high ENTPD8 expression may provide survival benefits to patients receiving immunotherapy. This also prompts us to further explore the relationship between ENTPD8 and immunity.

### Overexpression of ENTPD8 inhibits HCC growth and enhances anti-PD-L1 efficacy

**Figure 6:**
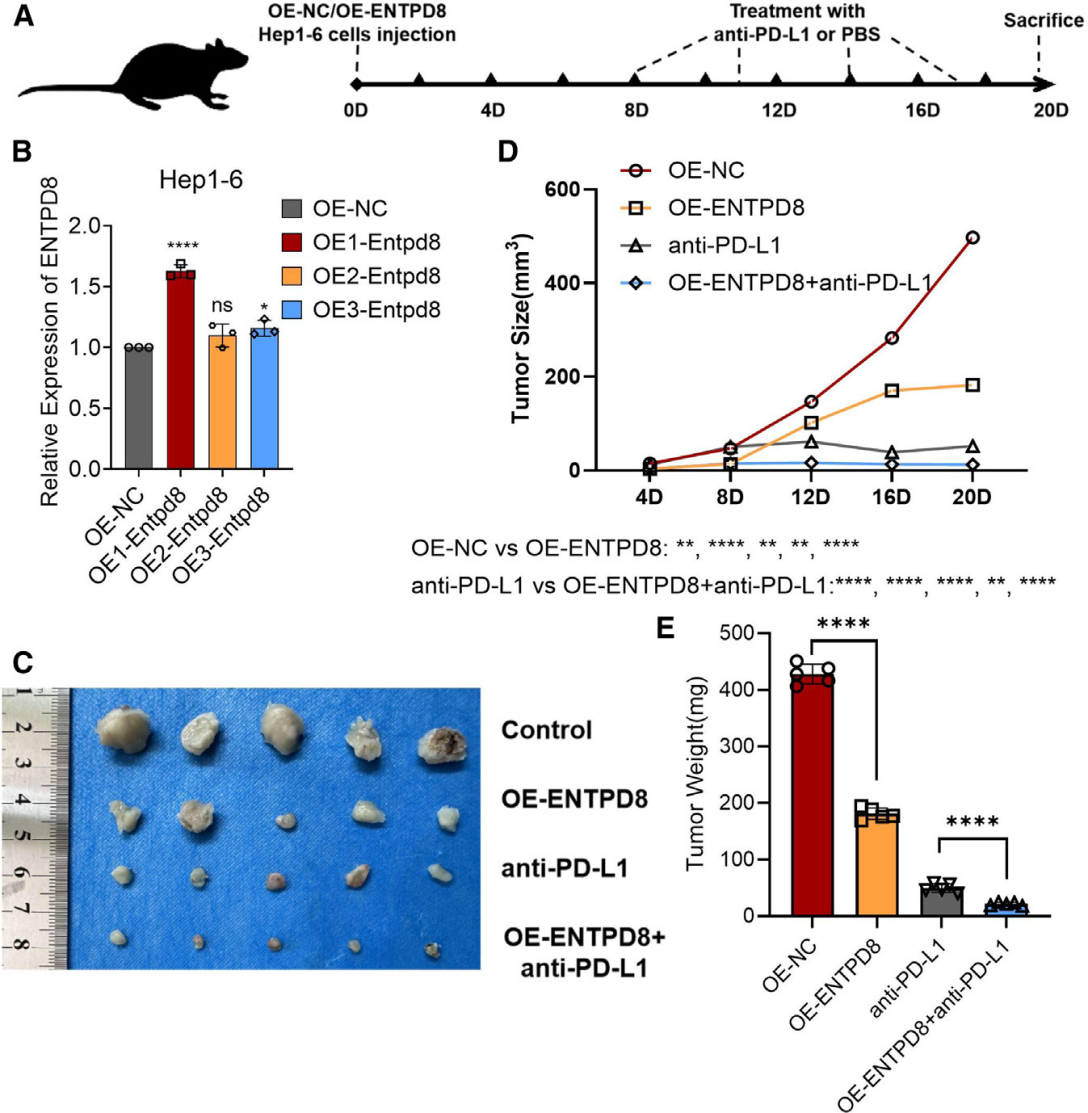
Overexpression of ENTPD8 inhibits HCC growth and enhances anti-PD-L1 efficacy (A) Schematic diagram of establishment of mouse subcutaneous tumor model in each group. (B) Three lentiviruses were designed to overexpress ENTPD8 expression in Hep 1–6 cell lines, and their overexpression efficiency was verified by qRT-PCR. (C) Images of subcutaneous tumors in each group. (D and E) Analysis of subcutaneous tumors in the respective groups. ∗, *p* < 0.05; ∗∗, *p* < 0.01; ∗∗∗, *p* < 0.001; ∗∗∗∗, *p* < 0.0001. Data are represented as mean ± SEM.

The aforementioned results suggest that high expression of ENTPD8 may promote the efficacy of anti-PD-L1 to some extent. To further validate the impact of ENTPD8 on the efficacy of anti-PD-L1 treatment, the *in vivo* experiments were conducted (Figure 6A). We synthesized three different lentiviral vectors to overexpress ENTPD8 in mouse HCC cell line Hep1-6. qRT-PCR analysis confirmed that the OE1-ENTPD8 vector achieved the highest level of ENTPD8 overexpression (Figure 6B). Subcutaneous tumor models were established using both normal and ENTPD8-overexpressing Hep1-6 cells in mice. The data showed a significant inhibition of tumor growth after overexpression of ENTPD8. Additionally, mice treated with ENTPD8 overexpression showed a significantly enhanced efficacy of anti-PD-L1 treatment compared to those receiving either ENTPD8 overexpression alone or anti-PD-L1 treatment alone (Figures 6C–6E). These findings underscore the potential of ENTPD8 to enhance the sensitivity of anti-PD-L1 treatment.

### ENTPD8 upregulates the expression of PD-L1 by regulating miR-214-5p

**Figure 7:**
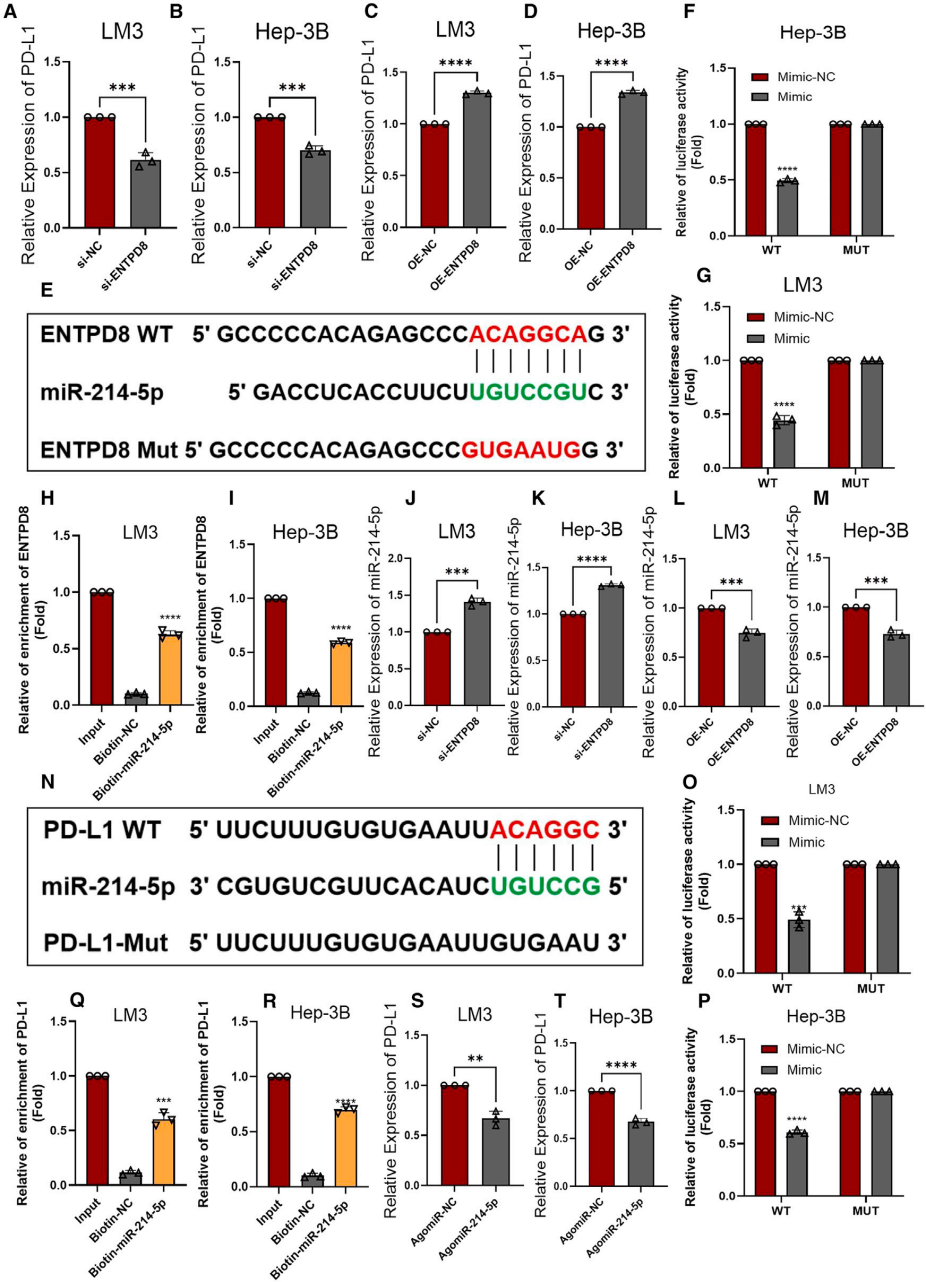
ENTPD8 up-regulates the expression of PD-L1 by regulating miR-214-5p (A–D) Changes in PD-L1 expression levels after knockdown (A and B) or overexpression (C and D) of ENTPD8 in HCC cell lines. (E) starBase predicted the binding sites of miR-214-5p and ENTPD8. (F and G) The association between miR-214-5p and ENTPD8 was confirmed by luciferase reporter gene assay. (H and I) RNA pull-down experiments were performed in Hep3B and LM3 cells. (J–M) Changes in miR-214-5p expression levels after knockdown (J and K) or overexpression (L and M) of ENTPD8 in HCC cell lines. (N) starBase predicted the binding sites of miR-214-5p and PD-L1. (O and P) The association between miR-214-5p and PD-L1 was confirmed by luciferase reporter gene assay. (Q and R) RNA pull-down experiments were performed in Hep3B and LM3 cells. (S and T) The expression level of PD-L1 was detected by qRT-PCR after the regulation of miR-214-5p. ∗, *p* < 0.05; ∗∗, *p* < 0.01; ∗∗∗, *p* < 0.001; ∗∗∗∗, *p* < 0.0001. Data are represented as mean ± SEM.

Building on aforementioned findings regarding the relationship between ENTPD8 expression levels and anti-PD-L1 treatment, we further investigated the connection between ENTPD8 and PD-L1. qRT-PCR results revealed that knocking down ENTPD8 reduces PD-L1 levels in HCC cells (Figures 7A, 7B, S3A, and S3C), whereas overexpressing ENTPD8 increases PD-L1 levels (Figures 7C, 7D, S3B, and S3D). This suggests that ENTPD8 may affect the efficacy of anti-PD-L1 treatment by modulating PD-L1 levels. Next, we focused on exploring the regulatory pathways of ENTPD8 on PD-L1 expression. We used starBase v.2.0 to explore miRNAs targeting ENTPD8. The data revealed a potential interaction between ENTPD8 and miR-214-5p (Figure 7E). Luciferase reporter gene assays showed that the addition of miR-214-5p reduced the luciferase activity of wild type (WT), but not PSMA3-AS1-mut (Figures 7F, 7G, and S3E), and RNA immunoprecipitation experiments demonstrated an interaction between ENTPD8 and miR-214-5p (Figures 7H, 7I, and S3F), suggesting that ENTPD8 may regulate the levels of miR-214-5p. Next, we knocked down or overexpressed ENTPD8 in LM3 and Hep3B cells, and then measured the levels of miR-214-5p by qRT-PCR. The results showed a negative correlation between ENTPD8 and miR-214-5p expression (Figures 7J–7M, S3G, and S3H). Previous studies have reported an interaction between miR-214-5p and PD-L1 in bladder cancer cells.^35^ Next, we verified their relationship in HCC. As shown in Figure 7N, the 3′ UTR of PD-L1 can bind to miR-214-5p. Luciferase reporter gene assays showed that the addition of miR-214-5p reduced the luciferase activity of PD-L1-wt, but not PD-L1-mut (Figures 7O, 7P, and S3I), and RNA pull-down experiments demonstrated an interaction between PD-L1 and miR-214-5p (Figures 7Q, 7R, and S3J). qRT-PCR results showed a significant decrease in PD-L1 expression levels after upregulating miR-214-5p in HCC cells (Figures 7S, 7T, and S3K). Finally, we enhanced ENTPD8 expression in LM3 and Hep3B cells and performed chromatin immunoprecipitationPCR; the result did not show that ENTPD8 binds to the promoter region of PD-L1 (Figures S3L and S3M). Based on the current data, ENTPD8 regulates PD-L1 expression through miR-214-5p in LM3, Hep3B and HepG2 cell lines, but further research is needed to validate the generalizability of this mechanism across other HCC cell lines.

### The expression of ENTPD8 was correlated with immune factors

**Figure 8:**
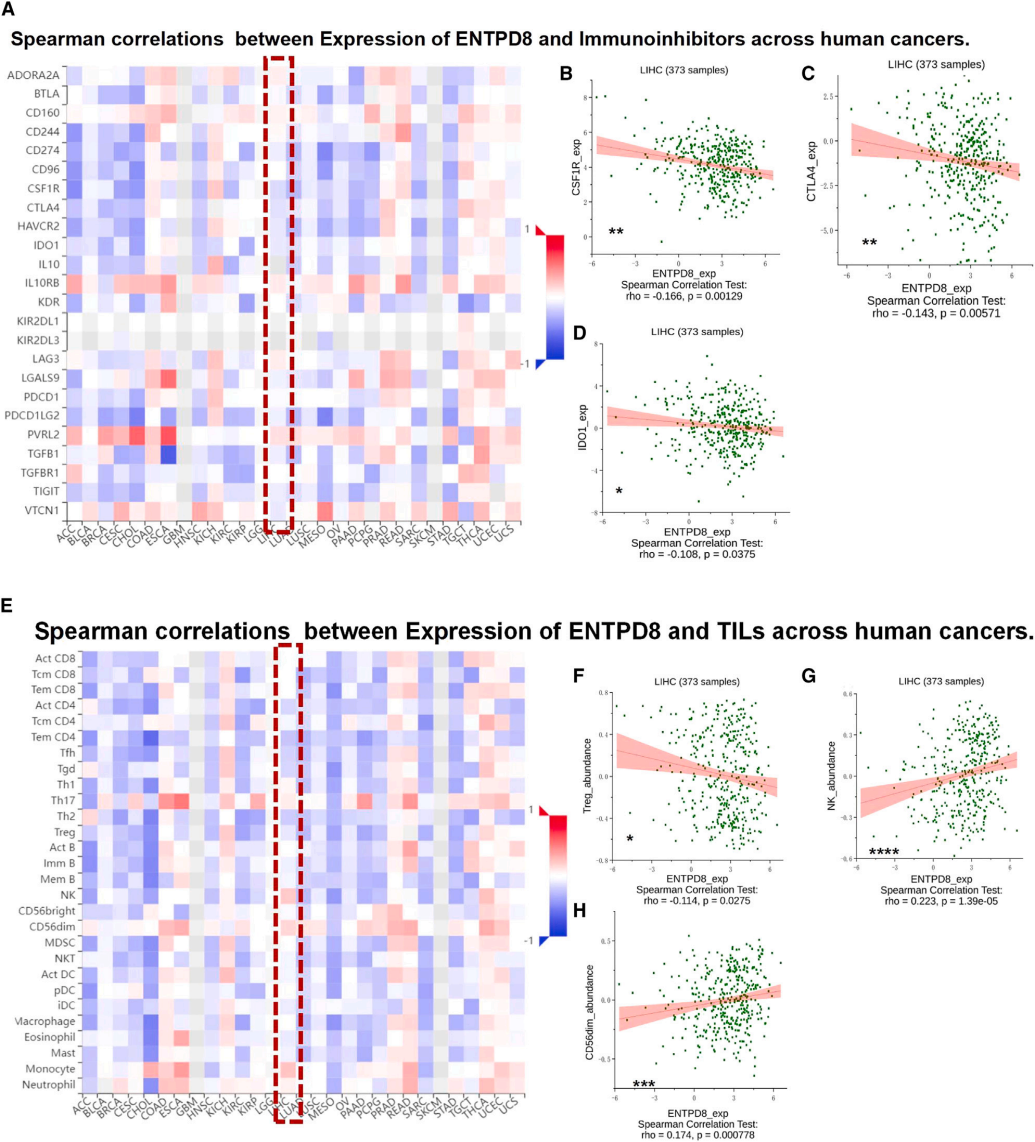
The expression of ENTPD8 was correlated with immunosuppressive factors (A) Correlation between ENTPD8 and immunoinhibitors in different cancers. (B–D) The relationship between the expression of ENTPD8 and the levels of immunoinhibitors CSF1R (B), CTLA4 (C), and IDO1 (D) in HCC. (E) Correlation between ENTPD8 and tumor-infiltrating lymphocytes (TILs) in different cancers. (F–H) The relationship between the expression of ENTPD8 and the levels of Treg (F), NK (G), and CD56dim (H) in HCC. ∗, *p* < 0.05; ∗∗, *p* < 0.01; ∗∗∗, *p* < 0.001; ∗∗∗∗, *p* < 0.0001.

The aforementioned results prompt us to consider whether ENTPD8 has a regulatory effect on the tumor immune microenvironment. We analyzed the relationship between ENTPD8 and various immune-related factors using the TISIDB database. Figure 8A illustrates the relationship between ENTPD8 and various immunoinhibitors in different cancer types. In HCC, ENTPD8 expression shows a negative correlation trend with CSF1R (Figure 8B), CTLA4 (Figure 8C), and IDO1 (Figure 8D) expression. Additionally, Figure 8E demonstrates the relationship between ENTPD8 and various immune cells in different cancer types. In HCC, ENTPD8 expression is negatively correlated with Treg cell levels (Figure 8F) and positively correlated with natural killer (NK) cells and CD56dim cells (Figures 8G and 8H). Figure S4 shows the relationship between ENTPD8 and various immune stimulatory factors in different cancer types.

## Discussion

HCC remains one of the most challenging malignancies globally, characterized by limited therapeutic options and poor prognosis. Hence, the identification of effective therapeutic targets is crucial. In our study, we identified ENTPD8 as a potential therapeutic target for HCC. Under physiological conditions, ENTPD8 primarily regulates extracellular nucleotide levels by hydrolyzing extracellular nucleotides and nucleoside triphosphates. Additionally, ENTPD8 is involved in biological processes such as cell adhesion, apoptosis, immune response, and cell migration.^8^^,^^9^^,^^10^^,^^12^^,^^13^ In the field of oncology, only a few studies have reported on the role of ENTPD8. Some research using metabolomics and transcriptomics has found alterations in the cytidine triphosphate (CTP) dephosphorylation pathway in peritumoral cirrhotic tissue (PCT) compared to adjacent non-cancerous tissue. ENTPD8 is downregulated in PCT, leading to reduced cytidine formation and thereby weakening CTP dephosphorylation in pyrimidine metabolism.^36^ Additionally, another study suggests that ENTPD8 could serve as a potential prognostic risk indicator in rectal cancer.^12^ However, the role of ENTPD8 in HCC has not yet been reported. Our study found that ENTPD8 expression is significantly downregulated in HCC tissues and shows a negative correlation with the mortality risk of patients with HCC. At the single-cell level, ENTPD8 is primarily expressed in tumor cells of HCC. Our *in vitro* experiments demonstrated that knocking down ENTPD8 promotes the proliferation, invasion, and migration of HCC cell lines, whereas overexpressing ENTPD8 in HCC cell lines has the opposite effect. These results suggest that ENTPD8 may play an anti-cancer role in HCC. Additionally, we were surprised to find that patients with HCC with high ENTPD8 expression exhibited better survival rates.

PD-L1 is an immune checkpoint molecule primarily expressed on the surface of tumor cells, immune cells, and non-tumor cells. Its main function is to inhibit the activation and proliferation of T cells by binding to PD-1 on the surface of T cells, thereby suppressing the attack of immune cells on tumor cells. The expression levels of PD-L1 are typically regulated by various factors, including tumor type, degree of immune cell infiltration, and levels of inflammatory factors.^17^^,^^18^^,^^19^ In recent years, PD-L1 has emerged as a crucial target for tumor immunotherapy and has been widely applied in the treatment of various tumors, such as non-small cell lung cancer, melanoma, renal cell carcinoma, and gastric cancer, achieving certain clinical efficacy.^22^^,^^24^^,^^25^ However, although PD-L1 monoclonal antibodies show significant efficacy in some patients, not all patients respond effectively to treatment, and a certain proportion of patients develop resistance to therapy. Mechanisms of resistance may include changes in tumor cell PD-L1 expression, activation of tumor immune escape pathways, and increased levels of other immune inhibitory factors in the tumor microenvironment. Therefore, a comprehensive understanding of PD-L1 is crucial.

Our study preliminarily discovered using the Kaplan-Meier plotter tool that patients with high ENTPD8 expression responded better to anti-PD-L1 treatment. However, it is important to note that these results have limitations, as high ENTPD8 expression alone can benefit the survival of patients with HCC. This suggests the need to explore the relationship between ENTPD8 and PD-L1. Our findings indicate that ENTPD8 can upregulate PD-L1 expression, suggesting that high ENTPD8 expression can enhance the efficacy of anti-PD-L1 treatment. Additionally, our *in vivo* experiments further confirmed that the combined overexpression of ENTPD8 and anti-PD-L1 can effectively inhibit HCC progression. The anti-cancer effects of the combined therapy were significantly superior to those of the monotherapy. These results are encouraging and suggest that targeting ENTPD8 as a therapeutic strategy for HCC has great potential.

MicroRNAs (miRNAs) are a class of short non-coding RNAs that play important roles in gene regulation and are often dysregulated in cancer.^37^^,^^38^ Some classical miRNAs, such as miR-21, miR-155, miR-10b, and the miR-200 family, are considered the most common miRNAs involved in tumor initiation and progression.^39^^,^^40^^,^^41^^,^^42^^,^^43^^,^^44^^,^^45^^,^^46^^,^^47^ miR-214-5p has been found to be involved in tumor progression and metastasis in various cancers. Previous studies have suggested that miR-214-5p may serve as an early biomarker for tracking the progression of liver fibrosis to HCC.^48^ Research has also found that the expression of miR-214-5p is significantly lower in HCC tissues compared to normal control tissues, indicating its potential role as a suppressor in hepatocellular carcinoma development and progression.^49^ Overexpression of miR-214-5p significantly inhibited HCC cell migration and invasion, while inhibition of miR-214-5p promoted migration and invasion. Furthermore, miR-214-5p suppressed epithelial-mesenchymal transition. Further studies identified WASL and KLF5 as potential target genes of miR-214-5p. Upregulation of WASL expression could reverse the inhibitory effects of miR-214-5p on invasion and migration.^50^^,^^51^ Additionally, miR-214-5p, along with miR-409-3p, can be sequestered by LINC00886, which in turn regulates the upregulation of RAB10 and E2F2 and promotes HCC development by activating the nuclear factor κB pathway. This suggests that the LINC00886-miR-409-3p/miR-214-5p axis plays a critical role in HCC.^52^ In our research, we found that ENTPD8 may regulate the expression of miR-214-5p, thereby affecting the expression of PD-L1 and enhancing the sensitivity of HCC cells to PD-L1 therapy. We believe that this mechanism is not inconsistent with the anti-cancer effects of miR-214-5p in some specific contexts, because the target genes and regulatory effects of miR-214-5p may be different in different cellular environments. Therefore, the anti-cancer effect of ENTPD8 is not dependent on miR-214-5p, and it can affect tumor immunity by regulating the expression of miR-214-5p, rather than directly through the anti-tumor effect of miR-214-5p. We will explore the mechanism in more depth in future studies.

Despite significant advancements in the treatment of HCC, the prognosis for many patients remains poor due to late-stage diagnosis and limited therapeutic options. In this study, we have identified ENTPD8 as a promising biomarker and therapeutic target in HCC. However, several knowledge gaps remain that warrant further exploration. First, while ENTPD8 overexpression was shown to inhibit HCC cell proliferation, migration, and invasion, and enhance anti-PD-L1 therapy, the precise molecular mechanisms by which ENTPD8 regulates these processes, particularly through its interaction with miR-214-5p and PD-L1, require deeper investigation. Understanding the full extent of ENTPD8’s role in immune modulation and tumor progression could reveal additional therapeutic avenues and help to define its potential as a predictive biomarker for response to immunotherapy.

Moreover, although our results suggest that ENTPD8 could be a key factor in improving the efficacy of anti-PD-L1 therapy, this study was primarily based on preclinical models. Future research should aim to validate these findings in clinical cohorts to assess the clinical relevance and safety of ENTPD8-based strategies, potentially in combination with immune checkpoint inhibitors like anti-PD-L1. Furthermore, it is important to explore whether ENTPD8’s effects are cancer type specific or if its therapeutic potential can be generalized to other malignancies.

The identification of ENTPD8 as a therapeutic target also highlights the broader challenge in cancer immunotherapy: the need to identify reliable biomarkers and develop combination strategies that can overcome immune resistance. As the field of immuno-oncology continues to evolve, we anticipate that further research into the molecular mechanisms of ENTPD8, particularly its role in immune modulation, will play a critical part in optimizing the treatment strategies for HCC and potentially other cancers. The integration of ENTPD8 into combination therapy approaches may represent an exciting frontier in improving treatment outcomes for patients with cancer.

### Limitations of the study

Although *in vitro* and *in vivo* models showed promising results, they cannot fully replicate the complexity of the human tumor microenvironment. The mechanisms underlying ENTPD8 regulation of PD-L1 remain incompletely understood, and further investigation is needed. Additionally, while ENTPD8 shows potential as a therapeutic target, its clinical feasibility and safety in humans require validation through clinical trials. Despite these limitations, our findings suggest that ENTPD8 could serve as a valuable prognostic marker and therapeutic target for HCC.

## Resource availability

### Lead contact

Further information and requests for resources and reagents should be directed to the lead contact, Zhengwei Song (doctorsongzw@zjxu.edu.cn).

### Materials availability

This study did not generate new unique reagents.

### Data and code availability

• Single-cell sequencing data have been deposited at the Gene Expression Omnibus and are publicly available as of the date of publication. The accession numbers GSE125449, GSE146115, and GSE166635 are listed in the key resources table.
• This article does not report the original code.
• Any additional information required to reanalyze the data reported in this article is available from the lead contact upon request.

## Acknowledgments

This research was supported by the Medical Health Science and Technology Project of Zhejiang Provincial Health Commission (2022KY1246) and the 10.13039/501100019976Science and Technology Bureau of Jiaxing City (2023AZ31002 and 2022AZ10009).

## Author contributions

S.-q.Z.: software, methodology, and formal analysis. M.-j.C. and F.C.: data curation and methodology. S.-q.Z., Z.-w.S., M.-j.C., and F.C.: writing – original draft. Z.-f.G., X.-p.L., L.-y.H., H.-y.C., S.-q.Z., and J.-y.X.: validation, visualization, and investigation. J.-y.X., J.-g.F., Z.-w.S., and S.-q.Z.: conceptualization, project administration, supervision, and writing – review and editing.

## Declaration of interests

The authors declare no competing interests.

## STAR★Methods

### Key resources table

**Table:** 

REAGENT or RESOURCE	SOURCE	IDENTIFIER
**Antibodies**		

Anti-PD-L1	Selleck	Cat# A2115; RRID: AB_3675704
ENTPD8 Antibody	Affinity Biosciences	Cat# DF14941; RRID: AB_3675705

**Chemicals, peptides, and recombinant proteins**		

PBS	KeyGEN	KGL2206-500
DMEM	KeyGEN	KGL1206-500
RPMI-1640	KeyGEN	KGM31800-500
FBS	KeyGEN	KGL3001-50
Cell Counting Kit-8	DOJINDO	Lot. PH623

**Critical commercial assays**		

Tiangen RNA extraction kit	TIANGEN	DP451
BlasTaq™ 2X qPCR MasterMix	Abm	Cat. No. G891, G892
All-In-One 5X RT MasterMix	Abm	Cat. No. G592
Hyperactive pG-MNase CUTandRUN Assay Kit for PCR/qPCR	Vazyme	HD101-01

**Deposited data**		

Single cell sequencing data	Gene Expression Omnibus	GSE125449, GSE146115 and GSE166635

**Experimental models: Cell lines**		

Hepa 1-6	ATCC	CRL-1830
Hep3B	ATCC	HB-8064
Hep G2	ATCC	HB-8065
HCCLM3	CAS	TCHu270

**Experimental models: Organisms/strains**		

C57BL/6 mouse	Gempharmatech	E2304250112

**Oligonucleotides**		

Human ENTPD8 qPCR primer Forward	GCCTCACGGCACTCATTCTC	N/A
Human ENTPD8 qPCR primer Reverse	CGCATCAAACACGATCCCAA	N/A
Mouse ENTPD8 qPCR primer Forward	TATACCTCTGACCCGACACAG	N/A
Mouse ENTPD8 qPCR primer Reverse	GCACCCCAAAAATCCACAGGA	N/A
PD-L1 qPCR primer Forward	TGGCATTTGCTGAACGCATTT	N/A
PD-L1 qPCR primer Reverse	TGCAGCCAGGTCTAATTGTTTT	N/A
ENTPD8 siRNA#1	KeyGEN	N/A
ENTPD8 siRNA#2	KeyGEN	N/A
ENTPD8 siRNA#3	KeyGEN	N/A
ENTPD8 OE#1	KeyGEN	N/A
ENTPD8 OE#2	KeyGEN	N/A
ENTPD8 OE#3	KeyGEN	N/A
miR-214-5p mimic	KeyGEN	N/A
Biotin-miR-214-5p	KeyGEN	N/A
AgomiR-214-5p	KeyGEN	N/A

**Software and algorithms**		

ImageJ	Open source	N/A
GraphPad Prism	GraphPad	https://www.graphpad.com/
TCGAportal	https://www.cancer.gov/tcga	N/A
UALCAN	http://ualcan.path.uab.edu/	N/A
TISCH2	http://tisch.comp-genomics.org/	N/A
The Kaplan-Meier Plotter platform	http://kmplot.com/analysis/	N/A
Human Protein Atlas	https://www.proteinatlas.org/	N/A
TISIDB	http://cis.hku.hk/TISIDB/index.php	N/A

### Experimental model and study participant details

This study involved human participants and animal experiments were approved by the Ethics Committee of the Second Affiliated Hospital of Jiaxing University (JUMC2023-009). All laboratory procedures and animal care are guided by institutional ethical guidelines for animal-related laboratory procedures and are conducted in accordance with guidelines for the humane use and care of laboratory animals. Written informed consent was obtained from all participants. Tissue specimens were collected from three HCC patients who underwent partial hepatectomy between May 2020 and June 2023. After collection, all tissues were stored at -80°C for future use.

### Method details

#### Ethics approval and consent to participate

This study involves human participants and the animal experiment were approved by the Ethics Committee of the Second Affiliated Hospital of Jiaxing University. All experiment procedures and animal caring were in accordance with the institutional ethics directions for animals-related experimental processes. Written informed consent was obtained from all participants.

#### Analysis of ENTPD8 expression

The resources from TCGAportal (https://www.cancer.gov/tcga) and UALCAN (http://ualcan.path.uab.edu/) were harnessed to study variations in ENTPD8 expression in non-cancerous tissues near tumors as well as in cancerous samples. UALCAN is a comprehensive, user-friendly, and interactive web resource for analyzing cancer OMICS data.^53^^,^^54^ Furthermore, these databases were instrumental in comparing ENTPD8 levels in HCC patients, considering factors such as disease stage and grade, race, gender, age, and weight. The TISCH database (http://tisch.comp-genomics.org/) is also a valuable resource for evaluating the association between ENTPD8 expression and the risk of various cancers.

#### Cell culture and transfection

Cells were cultured in RPMI 1640 Medium (KeyGen, China) supplemented with 10% fetal bovine serum (FBS) (KeyGen, China) at 37°C with 5% CO2. Penicillin (100 IU/mL) and streptomycin (100 mg/mL) were added to prevent microbial contamination. Custom-synthesized small interfering RNAs (siRNAs) targeting ENTPD8 (si-ENTPD8) and a non-targeting control siRNA (si-NC) were obtained from KeyGEN BioTECH (Jiangsu, China) for gene silencing. Transfection was performed using Lipofectamine 2000 (Invitrogen, USA) in Opti-MEM medium (Gibco, USA). Cells were harvested 48 hours post-transfection for further experiments. The specific sequences targeted by si-ENTPD8 were: 5'-CCACAGACATCAAGTTTGGGATCGT-3', 5'-CAGACATCAAGTTTGGGATCGTGTT-3', and 5'-GACATCAAGTTTGGGATCGTGTTTG-3'. A lentivirus engineered to overexpress ENTPD8 was obtained from KeyGEN BioTECH (Jiangsu, China) for both *in vitro* and *in vivo* experiments. Cells were initially plated at a density of 1×10^5^ cells per well in a 6-well plate and incubated with 2 mL of media for 24 hours. Following this, the media was replaced with 1 mL of fresh media containing the lentivirus and 40 μL of polybrene (Sigma, USA), and cells were incubated for 12-16 hours before returning to normal growth medium. Transfection efficiency was assessed using qRT-PCR.

#### qRT-PCR

Following the established protocol, we successfully isolated total RNA using the Tiangen RNA extraction kit (Tiangen, China). Gene expression was quantified with the BlasTaq™ 2X qPCR MasterMix(abm, China). Glyceraldehyde 3-phosphate dehydrogenase (GAPDH) was used as the reference gene for normalization. The expression data were analyzed using the relative quantification 2−ΔΔCt method. For the amplification of human ENTPD8, we used the following primer sequences: Forward Primer: 5'-GCCTCACGGCACTCATTCTC-3', Reverse Primer: 5'-CGCATCAAACACGATCCCAA-3'. Similarly, mouse ENTPD8 amplification was facilitated with primers: Forward Primer: 5'-TATACCTCTGACCCGACACAG-3', Reverse Primer: 5'-GCACCCCAAAAATCCACAGGA-3'.

#### Cell proliferation assay

Hep3B and LM3 cells were carefully divided into experimental and control groups and plated separately in 96-well plates. Each well received 1,000 cells suspended in 100 μL of growth medium. Treatment began with the addition of 10 μL of CCK-8 solution (DOJINDO, China). Optical density at 450 nm was recorded using a microplate reader (Synergy, USA) at the start of the culture and then at 24, 48, and 72 hours, following the manufacturer's operational protocols.

#### Transwell migration and invasion assays

In our assay setup, we added 200 μL of serum-deprived RPMI 1640 medium to the upper chamber and 700 μL of RPMI 1640 supplemented with 10% fetal bovine serum to the lower chamber. Each chamber received 20,000 cells. To assess cell invasiveness, we coated the Transwell insert (Corning, USA) with Matrigel (BD Biosciences, USA), which was not used for migration assessment. After 24 hours of incubation, non-invasive cells in the upper chamber were removed, and the medium was aspirated. The cells were then fixed with 4% paraformaldehyde for a brief period, stained with crystal violet (Kagan, China) for 15 minutes, and rinsed with PBS. Microscopic examination and enumeration of the cells were subsequently performed.

#### Clone forming assays

Both unmodified and modified cells were seeded into a 6-well plate at a density of 1,000 cells per well and cultured in RPMI 1640 supplemented with 10% fetal bovine serum. After a 10-day incubation period, the cells were fixed with methanol and stained with Giemsa. The colonies were then imaged and counted.

#### Survival analysis

The Kaplan-Meier Plotter platform (accessible at http://kmplot.com/analysis/) provides a powerful tool for investigating the relationship between gene expression (including mRNA, miRNA, protein, and DNA) and survival outcomes in over 35,000 samples from 21 cancer types. We utilized this database to assess the impact of ENTPD8 expression on the prognosis of HCC patients. The public datasets involved are: GSE31384, GSE10694, GSE6857, GSE20017, GSE9843 and TCGA. The "Auto select best cutoff" function of the tool is used to divide the high expression group and the low expression group. When this automatic partitioning function is used, the tool is able to automatically calculate all possible cutoff values between the upper and lower quartiles, using the best-performing threshold as the cutoff value. By generating Kaplan-Meier survival curves, we compared the survival outcomes of different groups of patients. To strengthen our analysis, we calculated hazard ratios and log-rank P-values, all within the 95% confidence intervals.

#### Single-cell analysis

To delve into the single cell expression profile and subcellular localization of ENTPD8 in HCC and normal liver tissue, we employed the Tumor Immune Single-cell Hub 2 (TISCH2) and Human Protein Atlas, accessible at http://tisch.comp-genomics.org/home/ and https://www.proteinatlas.org/.

#### Immune-related analysis

TISIDB (http://cis.hku.hk/TISIDB/index.php) is the integration of a variety of heterogeneous data types of portal, is applied to the analysis of the interaction of tumor and immune system.^55^ Here, we used it to analyze the spearman correlation between ENTPD8 expression and immunomodulators.

#### RNA pull-down assay

The binding relationship between miR-214-5p and ENTPD8 or PD-L1 was verified by RNA downscaling. The cells were treated with biotin-labeled miR-214-5p (Bio-miR-214-5p-Wt or BiomiR-214-5p-Mut) and its NC (Bio-NC) (KeyGen, China). The cell lysate was then incubated with streptavidin beads. The relative RNA enrichment was detected.

#### Luciferase reporter assay

The ENTPD8 promoter was subcloned into the pGL3 vector to construct a reporter gene, which was then transfected into T24 and 5637 cells. The wild-type (wt) or mutant (mut) ENTPD8 and PD-L1 reporter genes were subcloned into the pmirGLO vector (Promega). HCC cells were co-transfected with either NC simulators or miR-214-5p simulators using Lipofectamine 3000 (Invitrogen).

#### Animal models

The animal studies were thoroughly evaluated and approved by the Jiaxing University Ethics Committee, in accordance with animal ethics and welfare standards. In this study, twenty male C57BL/6 mice were randomly assigned to four specific groups: OE-NC, OE-ENTPD8, OE-NC+anti-PD-L1, and OE-ENTPD8+anti-PD-L1. A tumor model was established using 2×10^6^ Hep1-6 cells, which were injected subdermally into the mice from either the OE-NC or OE-ENTPD8 cell lines. Treatment began on day 8 after cell inoculation, with mice in the anti-PD-L1 groups receiving intraperitoneal injections of anti-PD-L1 at a dosage of 6.6 mg/kg. The remaining groups were given an equivalent volume of PBS. Treatments were administered every four days. Mice were monitored daily for general health, and tumor dimensions were measured regularly using vernier calipers to determine the long (a) and short (b) diameters. Tumor volume was calculated using the formula V=ab^2/2^. Measurements were taken every four days to track tumor growth. The experiment concluded on day 20, at which point the mice were euthanized, and tumor mass data were collected for comprehensive analysis.

#### Chromatin Immunoprecipitation(ChIP)

The ChIP assays were performed using the Hyperactive pG-MNase CUTandRUN Assay Kit for PCR/qPCR (Vazyme, China). Cell chromatin complexes were collected following the manufacturer's instructions and analyzed via qRT-PCR.

### Quantification and statistical analysis

Graph creation and statistical analyses were performed using Prism software (GraphPad Software). Differences between two groups were evaluated using either a non-parametric unpaired two-way t-test or a paired two-way t-test. For comparisons involving three or more groups from a single variable, one-way ANOVA was applied, followed by a Tukey post hoc test. When analyzing four groups with two variables, a two-way ANOVA was conducted, followed by Fisher’s least significant difference test for multiple comparisons. For the scRNA-seq violin plots, a Student’s t-test was used, with adjustments made using the Benjamini–Hochberg procedure. Statistical significance was defined as P < 0.05.
