# Supplementary material for: ENTPD8 overexpression enhances anti-PD-L1 therapy in hepatocellular carcinoma via miR-214-5p inhibition
Source: iScience. 2025 Jan 16;28(2):111819. doi: 10.1016/j.isci.2025.111819 (PMC11849663; doi:10.1016/j.isci.2025.111819)
Supplement: Document S1. Figures S1–S4 [file mmc1.pdf]

iScience, Volume *28*

Supplemental information

ENTPD8 overexpression enhances anti-PD-L1 therapy in hepatocellular carcinoma via miR-214-5p inhibition

Si-qi Zhao, Min-jie Chen, Fei Chen, Zhao-feng Gao, Xiao-ping Li, Ling-yu Hu, Hai-ying Cheng, Jin-yan Xuan, Jian-guo Fei, and Zheng-wei Song

Supplementary Figure 1

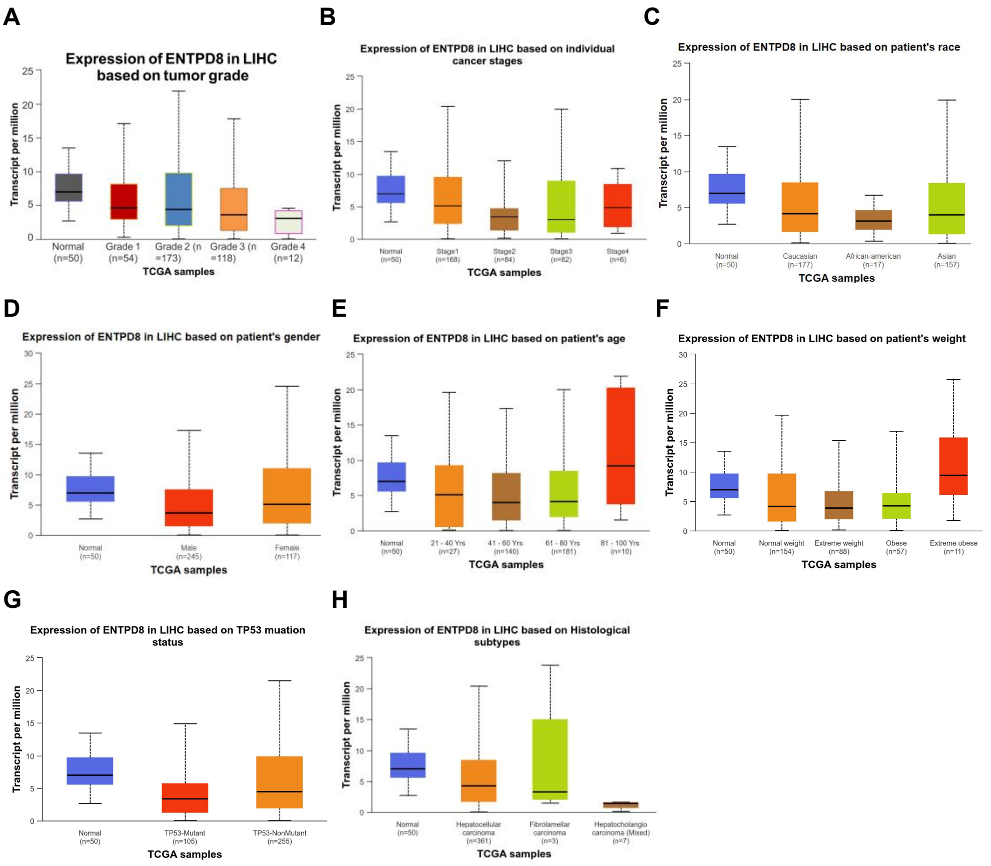

Figure S1. | Expression profile of ENTPD8 in HCC, related to Figure 1. (A) Relationship between ENTPD8 expression and tumor GRADE in HCC. (B) Relationship between ENTPD8 expression and individual cancer stage in HCC. (C) Relationship between ENTPD8 expression and patient's RACE in HCC. (D) Relationship between ENTPD8 expression and patient's GENDER in HCC. (E) Relationship between ENTPD8 expression and patient's AGE in HCC. (F) Relationship between ENTPD8 expression and patient's WEIGHTin HCC. (G) Relationship between ENTPD8 expression and TP53 muation status in HCC. (H) Relationship between ENTPD8 expression and Histological subtypes in HCC. Data are represented as mean ± SEM.

Supplementary Figure 2

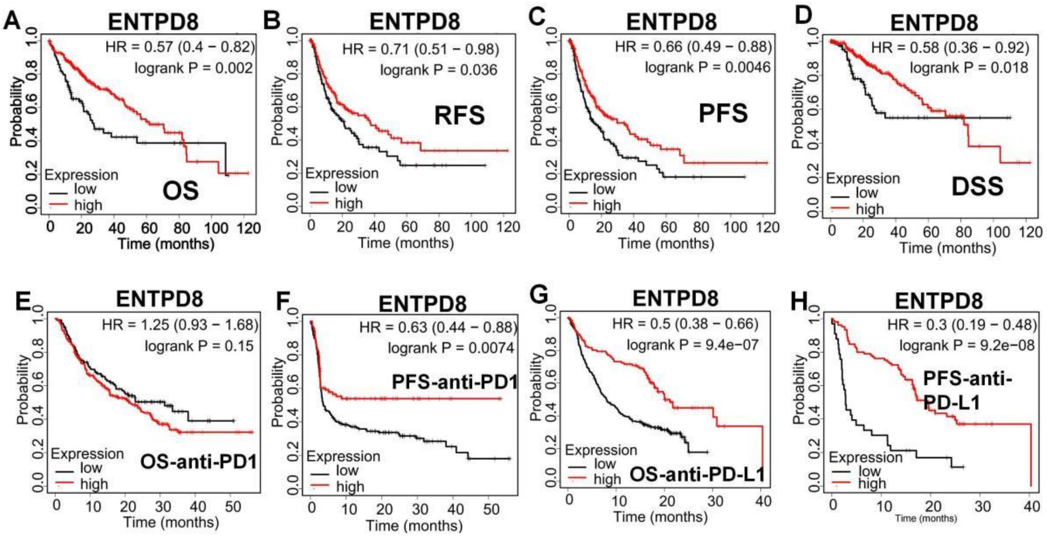

Figure S2. | ENTPD8 can be used as a prognostic indicator and a predictor of immunotherapy effect in HCC patients, related to Figure 6. (A-D) In HCC, patients with high expression of ENTPD8 had longer OS(A), RFS(B), PFS(C) and DSS(D) than those with low expression. (E) Relationship between ENTPD8 expression and OS in HCC patients treated with anti-PD1. (F)Relationship between ENTPD8 expression and PFS in HCC patients treated with anti-PD1. (G) Relationship between ENTPD8 expression and OS in HCC patients treated with anti-PD-L1. (H) Relationship between ENTPD8 expression and PFS in HCC patients treated with anti-PD-L1. *, P<0.05; **, P<0.01; ***, P<0.001;****, P<0.0001

Supplementary Figure 3

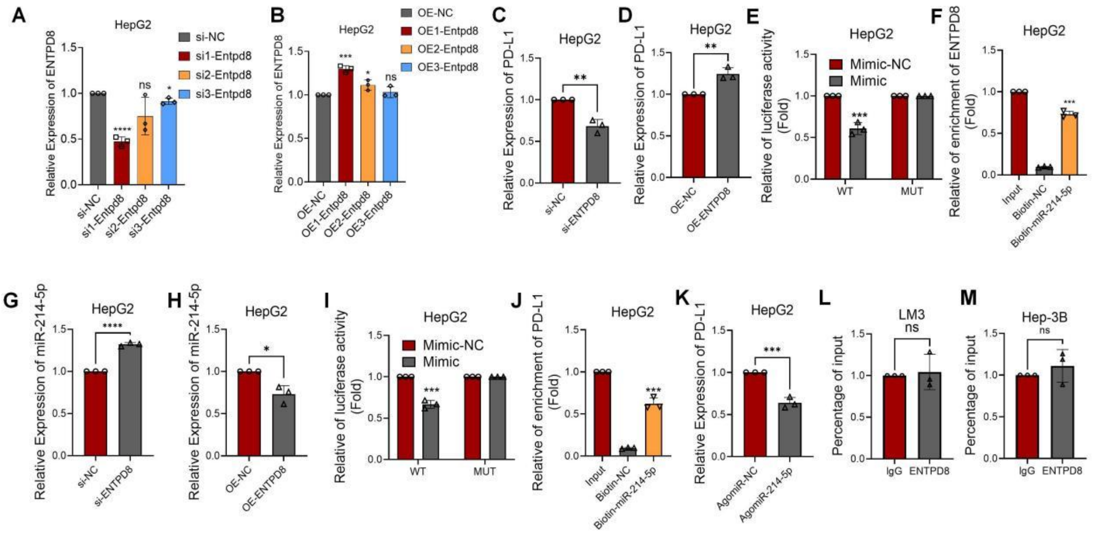

Figure S3. | ENTPD8 up-regulates the expression of PD-L1 by regulating miR-214-5p, related to Figure 7. (A-B) qRT-PCR verified the knockdown and overexpression efficiency of ENTPD8. (C-D) Changes in PD-L1 expression levels after knockdown(C) or overexpression(D) of ENTPD8 in HepG2. (E) The association between miR-214-5p and ENTPD8 was confirmed by luciferase reporter gene assay in HepG2. (F) RNA pull-down experiments were performed in HepG2 cells. (G-H) Changes in miR-214-5p expression levels after knockdown(G) or overexpression(H) of ENTPD8 in HepG2. (I) The association between miR-214-5p and PD-L1 was confirmed by luciferase reporter gene assay in HepG2. (J) RNA pull-down experiments were performed in HepG2. (K) The expression level of PD-L1 was detected by qRT-PCR after the regulation of miR-214-5p in HepG2. (L-M) ChIP analysis of ENTPD8 occupancy on PD-L1 promoters in LM3 and Hep3B cells. *, $P<0.05$; **, $P<0.01$; ***, $P<0.001$;****, $P<0.0001$. Data are represented as mean ± SEM.

Supplementary Figure 4

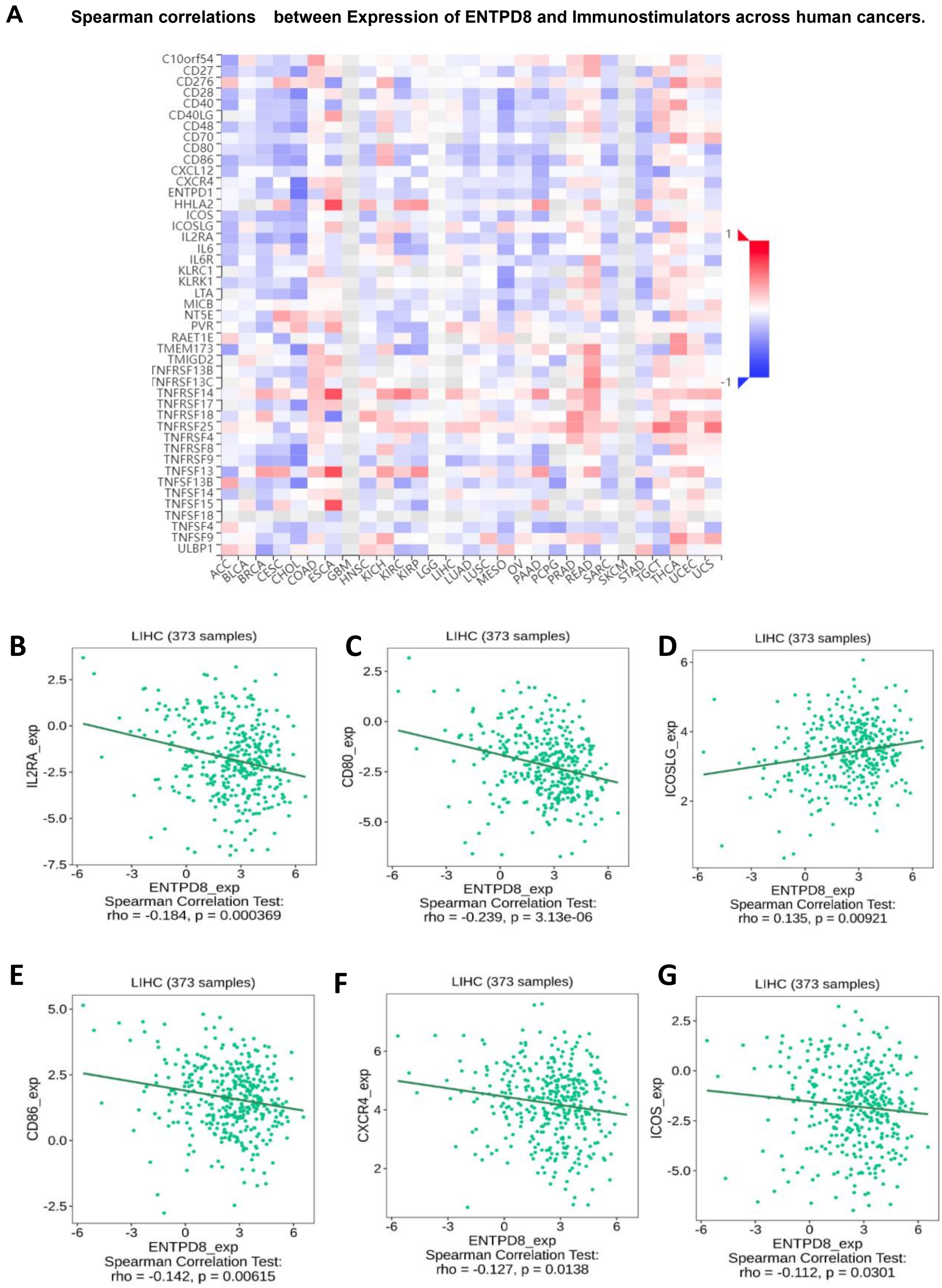

Figure S4. | The expression of ENTPD8 was correlated with immunostimulators, related to Figure 8. (A) Correlation between ENTPD8 and immunostimulators in different cancers. (B-G) The relationship between the expression of ENTPD8 and the levels of immunostimulators IL2RA (B), CD80 (C), ICOSLG (D), CD86 (E), CXCR4 (F) and ICOS(G) in HCC.
